# Pattern of Ureteric Pathology Presenting to a Fistula Centre in Western Kenya

**DOI:** 10.1155/2018/5056049

**Published:** 2018-04-24

**Authors:** Anthony Wanjala, Henry Mwangi, Hillary Mabeya

**Affiliations:** ^1^Malava County Hospital, Kakamega, P.O. Box 1561-50200, Bungoma, Kenya; ^2^Moi Teaching and Referral Hospital, P.O. Box 3-30100, Eldoret, Kenya; ^3^Department of Reproductive Health, Moi University School of Medicine, P.O. Box 3900-30100, Eldoret, Kenya

## Abstract

**Background:**

Ureteric pathology arises from surgical misadventures, trauma, and congenital anomalies. Early detection and treatment is of the essence.

**Objectives:**

To determine the types/etiology and outcome of ureteric pathology presenting to Gynocare Fistula Centre, Eldoret, Kenya.

**Methods:**

Descriptive retrospective study that evaluated patients presenting with ureteric pathology at Gynocare between 1st January 2012 and 31st December 2016. We pulled out patient charts and extracted and analyzed relevant data using STATA 13E statistical software.

**Results:**

We analyzed 33 charts, and their age ranged from 10 to 58 years. Annual proportion for 2012, 2013, 2014, 2015, and 2016 was 2.5%, 2.8%, 1.2%, 1.4%, and 3.0% respectively among all the fistula patients treated in the hospital. All the patients presented with urinary incontinence, and 7 (21.2%) had flank pain. Iatrogenic injuries contributed 84.8% (28), and 3 (9.1%) were congenital while trauma and infection had 1 each. Of those resulting from surgical misadventures, 17 (60.7%) were from obstetric while 11 (39.2%) were from gynecological surgery. All the injuries were in the distal third of the ureter; 5 were bilateral; and 11 were left sided while 17 were right-sided. Repair and/or reimplantation was successful in 31 (93.93%) of the patients.

**Conclusion:**

Highest proportion of ureteric pathologies was accounted for by iatrogenic causes and surgical repair and/or reimplantation has a high success rate.

## 1. Introduction

Ureteric pathology may arise from abdominal and pelvic surgery, trauma, congenital anomalies, malignancies, and radiation. Iatrogenic causes, though rare, account for most of the ureteric pathology [1]. The location of the ureter in the retroperitoneal space and its close proximity to the pelvic reproductive organs make it susceptible to injury during surgery [2]. Congenital anomalies of the ureter rarely occur alone and are likely to be accompanied by other anomalies on the urinary tree [3].

If not diagnosed and treated early, ureteric pathology is associated with worsening morbidity, including delayed hospital stay, risk of reoperation, compromise of the initial surgery, infection, ureterovaginal fistula, and loss of kidney function [4]. This is in addition to the psychological trauma felt by the patient and the possible risk of litigation. Patients may present with flank pain, urinary incontinence, fever, ascites, prolonged ileus, anuria, and even hydronephrosis and dysfunctional kidneys [5].

High index of suspicion and early diagnosis is of the essence in mitigating the effects of the resulting morbidities [6]. In the event of injury, several authors have demonstrated that early detection and treatment is associated with better short-term and long-term outcomes [2, 7].

In our set up, patients presenting with ureteric pathology are likely to be referred to a urologist if they are male, a fistula surgeon if they are female, and a pediatric surgeon in the event of children. Every so often, these subspecialties work together on select cases.

There exist gaps in published research on the extent of ureteric pathology in this region. The study set out to describe types/etiology and outcome of ureteric pathology presenting to a fistula centre in Western Kenya.

## 2. Methods

This was a descriptive retrospective study that evaluated all patients who presented with ureteric pathology at Gynocare Fistula Centre from 1st January 2012 to 31st December 2016. Located in Eldoret town on the Western part of Kenya, Gynocare is the biggest fistula referral centre in Kenya, with a bed capacity of 74. Every year the hospital performs over 300 fistula-related surgeries and manages many others conservatively. In 2014, the centre was accredited by WHO (World Health Organization) through FIGO (International Federation of Gynecology and Obstetrics) to train upcoming fistula surgeons. Since then, a total of 7 surgeons have been trained and certified while many others have visited to gain experience in specific areas.

The study population included all women with a diagnosis of ureteric pathology admitted at Gynocare center for a period of 5 years (from 1st January 2012 to 31st December 2016).

Patients' medical records were retrieved from the records department, and relevant data were extracted and entered on to chart review forms. This included information on bio-data, presenting symptoms, examination findings, diagnosis, presurgical evaluation, treatment offered, and condition at discharge. Presurgical evaluation included dye studies, kidney function tests, and abdominopelvic ultrasound. Where there was need, examination-under-anesthesia was done. Findings at surgery were also captured, including the type and location of ureteric pathology, comorbidities, and the surgical intervention chosen by the surgeon.

Data analysis was done using STATA 13E statistical software. Descriptive statistics were done to explore and summarize the data. Categorical data were summarized through frequency and proportions and presented in tables and charts, while numerical data were summarized using measures of central tendency and dispersion.

We sought approval from the Gynocare administration and scientific committee.

We used deidentifiers to code the review forms.

## 3. Results

### 3.1. Demographics

**Figure 1:**
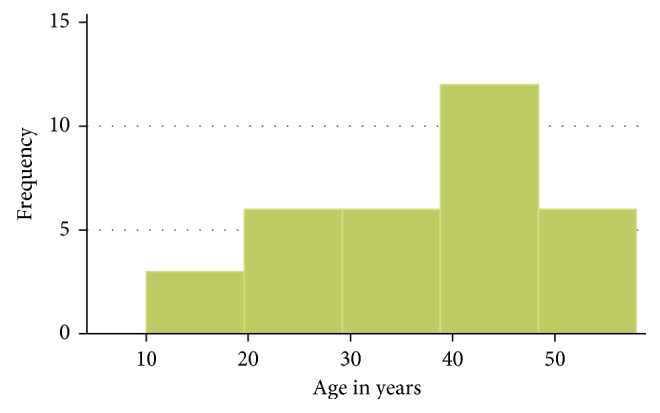
Age distribution.

A total of 33 patients were admitted to Gynocare with ureteric pathology between 1st January 2012 to 31st December 2016. All were female, and their age ranged from 10 years to 58 years with a mean of 36.8 (SD 12) years and a median of 41 (IQR 14). Peak age incidence was in the age range 40–49 years (Figure 1). Slightly above half 18 (54.6%) were referrals, where majority 10 (58.8%) of these were referred by community health workers and 7 (41.2%) were referred from other health facilities (Table 1). Median duration of symptoms was 0.25 years (IQR 8.9) with minimum being 0.019 (1 week) and maximum 17 years.

### 3.2. Presentation, Annual Proportion, and Etiology

All the patients complained of urinary incontinence at presentation while only 7 (21.2%) presented with flank pain (Table 1). Other complains included abdominal pain, amenorrhea, and per vaginal discharge.

The annual proportion of patients presenting with ureteric pathology against all the other fistula patients never exceeded 3% for the years 2012–2016, the highest, 3% coming in 2016 (Table 2). This is despite a steady rise in the number of fistula surgeries from 2012 to 2016.

At 84.8%, ureteric injuries from surgical misadventures contributed the highest percentage of pathologies, with obstetric accounting for 17 (60.7%) and gynecological surgeries accounting for 11 (39.3%) (Table 3). Of the remaining, 3 (9.1%) patients had congenital malformations while trauma and infection contributed 1 each. In the congenital malformation category, 2 had left sided double ureter with one opening through the vagina while the other had a normal right ureter opening into the urethra. All the pathologies were in the distal third of the ureter, 5 (15.2%) were bilateral, 11 (33.3%) left sided while 17 (51.5%) were right-sided.

### 3.3. Renal Function Tests

As a diagnostic and presurgical evaluation, all patients underwent a mandatory renal function and electrolyte test upon presentation. They were largely within the reference range, save for 1 (3.3%) who had elevated creatinine, urea and potassium and another who had elevated creatinine with normal urea and potassium (Table 4). Presurgical renal function tests for 3 patients could not be traced. Prior to discharge, the lab works were repeated and all were within reference ranges.

### 3.4. Management

Surgical intervention was the management of choice for all the 33 patients (Table 5). Slightly over half had ureteroneocystostomy while close to one-third had both ureteroneocystostomy and fistula repair. The remaining two patients had repair. Upon discharge, 2 patients still had urinary incontinence and were asked to come back for reoperation later. One presented to another fistula centre for repair while the other was lost to follow-up. Median time of hospitalization was 17 days (IQR 2), with minimum being 14 days and maximum 48 days. Postoperatively, there was no reported mortality or severe morbidity.

## 4. Discussion

Ureteric pathology can lead to serious morbidity and mortality if not detected early and treated. Among the leading causes of this is inadvertent ureteric injury during surgery [8, 9].

In the female pelvis, the ureter lies close to the uterus and the cervix making it susceptible to injury during either gynecological or obstetric surgeries [10]. As such, lower third ureteric injuries, just like in this study, are the commonest, probably from dissection of lower uterus and cervix or from injury during ligation of uterine vessels. It is thought that as high as 50–75% of these injuries arise from gynecological procedures [5], while the rest are largely from obstetric causes. This is well demonstrated in this study as about 89% of all ureteric pathology resulted from surgical misadventures, majority being obstetric (60%). Obstetric surgeries associated with ureteric injury include cesarean section, repair of uterine rupture, and cesarean hysterectomy. Advancement in labor management and monitoring has led to an increase in the CS rate the world over. In Kenya, most of these are done by relatively junior doctors due to acute shortage of specialists. This may account for the cases of ureteric injury from obstetric causes as less surgical experience is a risk factor to injury [11]. Gynecological procedures associated with ureteric injury include total abdominal hysterectomy, vaginal hysterectomy, removal of pelvic masses, oophorectomy, suspension procedures, and so on.

The annual proportion of 3% or less cannot be easily compared with other studies because they have used total hospital surgeries as the baseline whereas this study uses total fistula surgeries as the baseline, an obvious bias. Nevertheless with such annual proportion at a referral centre it is safe to say ureteric pathology is not common.

The fact that all patients presented with urinary incontinence points to a likelyhood of ureterovaginal fistula which in this case is protective to the kidneys. This may explain why for most patients their renal function tests were within reference ranges despite some having had the pathology for a long duration. Ureteric injury without incontinence may lead to leakage of urine into the peritoneum with attendant peritonitis or loss of kidney function in the event of ureteric ligation [6].

The left ureter is more liable to injury due to slight uterine rotation [12, 13]. This contrasts somewhat with this study where slightly more than half of patients had right-sided pathology, majority of who were iatrogenic.

Surgical intervention has proven to be key in the treatment of patients with ureteric pathology [1]. In this, high index of suspicion, early diagnosis, and timely treatment offer patients the best chance of recovery. The surgical options available include ureteroneocystostomy with or without psoas hitch and/or boari flap, ureteroneocystostomy, end to end anastomosis, primary repair when there is partial resection, and suture removal in the event of ligation among others. Surgery offered cure for 31 (94%) of patients in this study.

## 5. Conclusion

Iatrogenic injury is the commonest cause of ureteric pathology, from either obstetric or gynecological surgeries. The close association between reproductive organs and the ureter makes it more liable to injury. Surgical intervention has a high success rate.

## Figures and Tables

**Table 1:** Age, referral status, and clinical presentation.

Variable	Categories	Frequency	Percentage
Age	<20 years	3	9.1
	≥20 years	30	90.9
Referral	No	15	45.5
	Yes	18	54.5
Referral from	Other health facilities	7	38.9
	Community health workers	10	55.6
	Self-referral	1	0.056
Incontinence	No	0	0
	Yes	33	100
Flank pain	No	26	78.8
	Yes	7	21.2

**Table 2:** Ureteric pathology annual proportion.

Year	Frequency	Total fistula	Proportion (%)
2012	5	200	2.5
2013	8	285	2.8
2014	4	325	1.2
2015	5	348	1.4
2016	11	372	3.0
Total	33		

**Table 3:** Etiology of ureteric pathology.

Variable	Categories	Frequency	Percentage
Cause	Previous surgery	28	84.8
	Congenital	3	9.1
	Trauma	1	3.0
	PID	1	3.0
Surgery type	Gynecology	11	39.3
	Obstetric	17	60.7

**Table 4:** Renal function at admission.

Variable	Categories	Frequency	Percentage
Urea	<3.0	14	46.7
	3.00–8.39	15	50.0
	>8.39	1	3.3
K	3.40–5.10	29	96.7
	>5.10	1	3.3
Creatinine	44.00–133.00	28	93.3
	>133.00	2	6.7

**Table 5:** Treatment offered.

Variable	Categories	Frequency	Percentage
Was surgery offered	No	0	0
	Yes	33	100
Type of surgery done	Repair	2	6.1
	Ureteroneocystostomy	21	63.6
	Repair + ureteroneocystostomy	10	30.3
Discharge condition	Leaking urine	2	6.1
	Not leaking urine	31	93.9
